# Scanpath modeling and classification with hidden Markov models

**DOI:** 10.3758/s13428-017-0876-8

**Published:** 2017-04-13

**Authors:** Antoine Coutrot, Janet H. Hsiao, Antoni B. Chan

**Affiliations:** 1 CoMPLEX, University College London, London, UK; 2 Department of Psychology, The University of Hong Kong, Pok Fu Lam, Hong Kong; 3 Department of Computer Science, City University of Hong Kong, Kowloon Tong, Hong Kong

**Keywords:** Scanpath, Eye movements, Hidden Markov models, Classification, Machine-learning, Toolbox

## Abstract

How people look at visual information reveals fundamental information about them; their interests and their states of mind. Previous studies showed that scanpath, i.e., the sequence of eye movements made by an observer exploring a visual stimulus, can be used to infer observer-related (e.g., task at hand) and stimuli-related (e.g., image semantic category) information. However, eye movements are complex signals and many of these studies rely on limited gaze descriptors and bespoke datasets. Here, we provide a turnkey method for scanpath modeling and classification. This method relies on variational hidden Markov models (HMMs) and discriminant analysis (DA). HMMs encapsulate the dynamic and individualistic dimensions of gaze behavior, allowing DA to capture systematic patterns diagnostic of a given class of observers and/or stimuli. We test our approach on two very different datasets. Firstly, we use fixations recorded while viewing 800 static natural scene images, and infer an observer-related characteristic: the task at hand. We achieve an average of 55.9% correct classification rate (chance = 33%). We show that correct classification rates positively correlate with the number of salient regions present in the stimuli. Secondly, we use eye positions recorded while viewing 15 conversational videos, and infer a stimulus-related characteristic: the presence or absence of original soundtrack. We achieve an average 81.2% correct classification rate (chance = 50%). HMMs allow to integrate bottom-up, top-down, and oculomotor influences into a single model of gaze behavior. This synergistic approach between behavior and machine learning will open new avenues for simple quantification of gazing behavior. We release *SMAC with HMM*, a Matlab toolbox freely available to the community under an open-source license agreement.

## Introduction

We use vision to guide our interactions with the world, but we cannot process all the visual information that our surroundings provide. Instead, we sequentially allocate our attention to the most relevant parts of the environment by moving our eyes to bring objects onto our high-resolution fovea to allow fine-grained analysis. In natural vision, this endless endeavor is accomplished through a sequence of eye movements such as saccades and smooth pursuit, followed by fixations. These patterns of eye movements, also called *scanpaths*, are guided by the interaction of three main factors (Kollmorgen et al. 2010). First, *top-down* mechanisms are linked to the observers, and adapt their eye movements to their personal characteristics. They can be conscious like performing the task at hand, or unconscious like observers’ culture, age, gender, personality, or state of health. Second, *bottom-up* mechanisms are linked to the visual stimulus. They can be low-level such as local image features (motion, color, luminance, spatial frequency), or high-level such as the social context or the presence of faces and other semantic content. The third factor is related to the characteristics inherent to the *oculomotor system*, such as the spatial bias to the center region and the geometric properties of saccades.

### Gaze patterns contain a wealth of information

As a byproduct of these three multivariate mechanisms, eye movements are an exceptionally rich source of information about the observers and what they look at; they provide a high-resolution spatiotemporal measure of cognitive and visual processes that are used to guide behavior. Since the seminal work of Buswell and Yarbus (Buswell 1935; Yarbus 1965), several recent studies have proposed computational and statistical methods to infer observers’ characteristics from their eye movements. Since 2012, numerous studies have tried to classify observers’ gaze patterns according to the *task at hand* during reading (Henderson et al. 2013), counting (Haji-Abolhassani and Clark 2013), searching (Zelinsky et al. 2013), driving (Lemonnier et al. 2014), mind wandering (Mills et al. 2015), memorizing, and exploring static artificial or natural scenes (Kanan et al. 2014; Borji and Itti 2014; Haji-Abolhassani and Clark 2014). For a thorough review of task-prediction algorithms, see (Boisvert and Bruce 2016). Eye movements can also be used to quantify *mental workload*, especially during demanding tasks such as air traffic control (Ahlstrom and Friedman-Berg 2006; Di Nocera et al. 2006; Kang and Landry 2015; McClung and Kang 2016; Mannaru et al. 2016). Another very promising line of studies is gaze-based *disease screening* (Itti 2015). Eye movement statistical analysis is opening new avenues for quantitative and inexpensive evaluation of disease. Visual attention and eye movement networks are so pervasive in the brain that many disorders affect their functioning, resulting in quantifiable alterations of eye movement behavior. Both mental and eye disease diagnostics can be informed with gaze data. Mental disorders include Parkinson’s disease, attention deficit hyperactivity disorder, fetal alcohol spectrum disorder (Tseng et al. 2013), autism spectrum disorder (Wang et al. 2015), early dementia (Seligman and Giovannetti 2015) and Alzeihmer’s disease (Lagun et al. 2011; Alberdi et al. 2016). See (Anderson and MacAskill 2013) for a review of the impact of neurodegenerative disorders on eye movements. Eye tracking can also help diagnose eye diseases such as glaucoma (Crabb et al. 2014), age-related macular degeneration (Rubin and Feely 2009; Van der Stigchel et al. 2013; Kumar and Chung 2014), strabismus (Chen et al. 2015), and amblyopia (Chung et al. 2015). In many cases (particularly where patients/young infants cannot talk), this has the added advantage of bypassing verbal report. Given the prevalence of (sometimes subtle) health disorders, developing assessment methods that allow researchers to reliably and objectively test all ages could prove crucial for effective early intervention. Other studies have used eye movements to successfully *infer observers’ characteristics* such as their gender (Coutrot et al. 2016), age (French et al. 2016), personality (Mercer Moss et al. 2012) and level of expertise (e.g., novices vs. experts in air traffic control (Kang and Landry 2015), medicine (Cooper et al. 2009), and sports (Vaeyens et al. 2007). See (Gegenfurtner et al. 2011) for a meta-analysis). A complementary approach uses eye movements to extract information about what is being seen. For instance, machine learning approaches have been used to infer the valence (positive, negative, neutral) of static natural scenes (Tavakoli et al. 2015). The category of a visual scene (e.g., conversation vs. landscape) can also be determined from eye movements in both static (O’Connell and Watlher 2015) and dynamic (Coutrot and Guyader 2015) natural scenes.

### Capturing gaze information

**Fig. 1:**
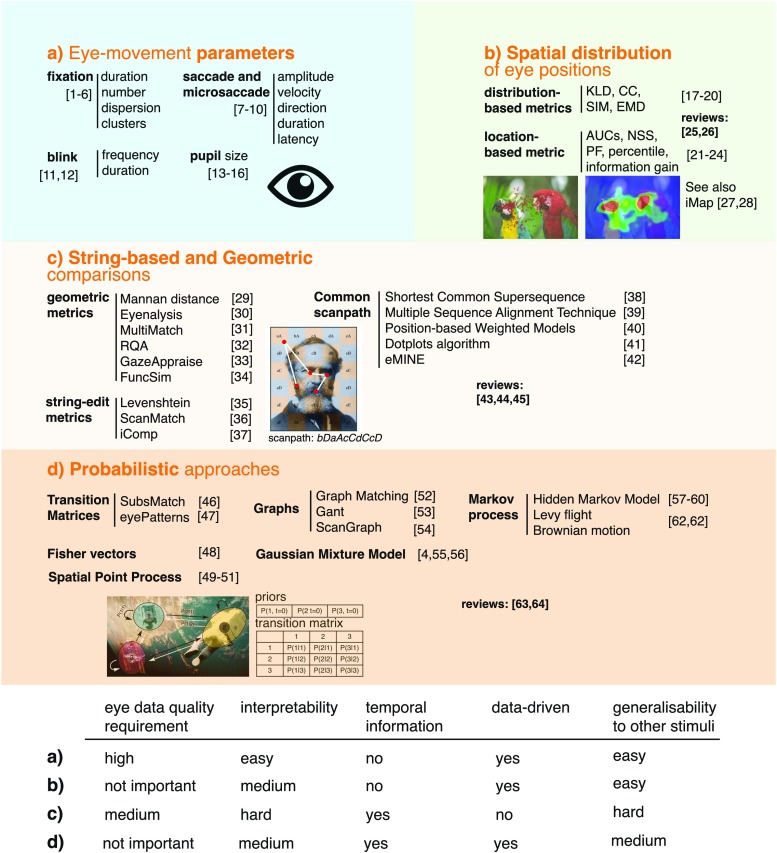
State-of-the-art in eye movement modeling and comparison. The different approaches are clustered into four groups: (a) Oculomotor parameters, (b) Spatial distribution of eye positions (KLD = Kullback-Leibler divergence, CC = correlation coefficient, SIM = similarity, EMD = earth moving distance, AUC = area under curve, NSS = normalized scanpath saliency, PF = percentage of fixation), (c) string-based and geometric scanpath comparisons, and (d) probabilistic approaches. Each technique is referenced, and relevant reviews are suggested. On the lower part, a table capsulizes the pros and cons of each type of approach: does it require high-quality eye data?; does it provide an easily interpretable model?; does it capture temporal information?; is it data-driven?; can it be applied to all types of stimuli? [1] Mills et al. (2015); [2] Lagun et al. (2011); [3] Tavakoli et al. (2015); [4] Mital et al. (2010); [5] Mills et al. (2011); [6] Kardan et al. (2015); [7] Le Meur & Liu (2015); [8] Le Meur & Coutrot (2016); [9] Martinez-Conde et al. (2009); [10] Ohl et al. (2016); [11] Ahlstrom & Friedman-Berg (2006); [12] Bulling et al. (2011); [13] Rieger & Savin-Williams, (2012); [14] Badnarik et al. (2012); [15] Wass & Smith (2014); Binetti et al. (2016); [17] Rajashekar et al. (2004); [18] Le Meur et al. (2006); [19] Toet (2011); [20] Judd et al. (2012); [21] Peters et al. (2005); [22] Torralba et al. (2006); [23] Peters & Itti (2008); [24] Kümmerer et al. (2015); [25] Riche et al. (2013); [26] Bylinskii et al. (2016); [27] Caldara & Miellet (2011); [28] Lao et al. (2016); [29] Mannan et al. (1996); [30] Mathôt et al. (2012); [31] Dewhurst et al. (2012); [32] Anderson et al. (2013); [33] Haas et al. (2016); [34] Foerster & Schneider, (2013); [35] Levenshtein (1966); [36] Cristino et al. (2010); [37] Duchowski et al. (2010); [38] Räihä (2010); [39] Hembrooke et al. (2006); [40] Sutcliffe & Namoun (2012); [41] Goldberg & Helfman (2010); [42] Eraslan et al. (); [43] Eraslan et al. (2016); [44] Le Meur & Baccino (2013); [45] Anderson et al. (2014); [46] Kübler et al. (2016); [47] West et al. (2006); [48] Kanan et al. (2015); [49] Barthelmé et al. (2013); [50] Engbert et al. (2015); [51] Ylitalo et al. (2016); [52] Rigas et al. (2012); [53] Cantoni et al. (2015); [54] Dolezalova & Popelka (2016); [55] Vincent et al. (2009); [56] Couronné et al. (2010); [57] Haji-Abolhassani & Clark (2014); [58] Coutrot et al. (2016); [59] Chuk et al. (2017); [60] Chuk et al. (2014); [61] Brockmann & Geisel (2000); [62] Boccignone & Ferraro (2004) [63] Boccignone (2015); [64] Galdi et al. (2016)

All the gaze-based inference and classification studies mentioned so far rely on a very broad range of gaze features. Gaze is a complex signal and has been described in a number of ways. In Figure 1, we review the main approaches proposed in the literature. Figure 1a takes an inventory of all eye movements direct parameters: *fixation* duration, location, dispersion, and clusters (Mital et al. 2010; Lagun et al. 2011; Mills et al. 2011; Kardan et al. 2015; Tavakoli et al. 2015; Mills et al. 2015), *saccade* amplitude, duration, latency, direction and velocity (Le Meur and Liu 2015; Le Meur and Coutrot 2016), *microsaccade* amplitude, duration, latency, direction and velocity (Martinez-Conde et al. 2009; Ohl et al. 2016), *pupil* dilation (Rieger and Savin-Williams 2012; Bednarik et al. 2012; Wass and Smith 2014; Binetti et al. 2016), *blink* frequency and duration (Ahlstrom and Friedman-Berg 2006; Bulling et al. 2011). The advantages of these features are their direct interpretability, the fact that they can be recorded on any stimulus set without having to tune arbitrary parameters (except saccade detection thresholds). Their drawbacks are that high-quality eye data is required to precisely parse fixations and saccades and measure their parameters, with a sampling frequency above 60 Hz (Nyström and Holmqvist 2010). Moreover, they are synchronic indicators: the events they measure occur at a specific point in time and do not capture the spatio-temporal aspect of visual exploration. In Fig. 1b, authors introduce spatial information with eye position maps, or heatmaps, which are three-dimensional objects (x, y, fixation density) representing the spatial distribution of eye positions at a given time. They can be either binary or continuous, if smoothed with a Gaussian filter. Different metrics have been proposed to compare two eye position maps and are either distribution-based: the Kullback–Leibler divergence (KLD) (Rajashekar et al. 2004), the Pearson (Le Meur et al. 2006) or Spearman (Toet 2011) correlation coefficient (CC), the similarity and the earth moving distance (EMD) (Judd et al. 2012); or location-based: the normalized scanpath saliency (NSS) (Peters et al. 2005), the percentage of fixation into the salient region (PF) (Torralba et al. 2006), the percentile (Peters and Itti 2008) and the information gain (Kümmerer et al. 2015). Most of these have been created to compare ground-truth eye position maps with visual saliency maps computed from a saliency model. Eye position maps are easy to compute with any stimuli; they only require simple (*x*,*y*) gaze coordinates. For instance, iMap is a popular open-source toolbox for statistical analysis of eye position maps (Caldara and Miellet 2011; Lao et al. 2016). As with eye movement parameters, this approach is mostly data-driven: only the size of the smoothing Gaussian kernel needs to be defined by the user. Eye position maps can be visually meaningful. However, each metric measures the distance between slightly different aspects of spatial distributions, which can be hard to interpret. We refer the interested reader to the following reviews: (Riche et al. 2013; Bylinskii et al. 2016). Their main drawback is that they fail to take into account a critical aspect of gaze behavior: its highly dynamic nature. To acknowledge that visual exploration is a chronological sequence of fixations and saccades, authors listed in Fig. 1c represent them as *scanpaths*. Different metrics have been proposed to compare two scanpaths. The simplest are string-edit distances (Levenshtein 1966; Cristino et al. 2010; Duchowski et al. 2010). They first convert a sequence of fixations within predefined regions of interest (or on a simple grid) into a sequence of symbols. In this representation, comparing two scanpaths boils down to comparing two strings of symbols, i.e., computing the minimum number of edits needed to transform one string into the other. More complex vector-based methods avoid having to manually predefine regions of interest by geometrically aligning scanpath (Mannan et al. 1996; Mathôt et al. 2012; Dewhurst et al. 2012; Anderson et al. 2013; Haass et al. 2016; Foerster and Schneider 2013) or finding common sequences shared by two scanpaths (Räihä 2010; Hembrooke et al. 2006; Sutcliffe and Namoun 2012; Goldberg and Helfman 2010; Eraslan *et al*. 2016). For instance, MultiMatch aligns two scanpaths according to different dimensions (shape, length, duration, angle) before computing various measures of similarity between vectors (Dewhurst et al. 2012). For further details, the reader is referred to the following reviews: (Le Meur and Baccino 2013; Anderson et al. 2014; Eraslan et al. 2016). The major drawback of both string-edit and geometric-based approaches is that they do not provide the user with an interpretable model of visual exploration, and often heavily rely on free parameters (e.g., the grid resolution). Figure 1d lists probabilistic approaches for eye movement modeling. These approaches hypothesize that eye movement parameters are random variables generated by underlying stochastic processes. The simplest gaze probabilistic model probably is Gaussian mixture model (GMM), where a set of eye positions is modeled by a sum of two-dimensional Gaussians. If the stimulus is static, eye positions can be recorded from the same observer and added up through time (Vincent et al. 2009; Couronné et al. 2010). They can also be recorded from different observers viewing the same stimulus at a given time (Mital et al. 2010). Modeling gaze with GMM allows to take into account fixations slightly outside regions of interest, considering phenomena such as the dissociation between the center of gaze and the covert focus of attention, the imprecision of the human oculomotor system and of the eye-tracker. However, the main advantage of statistical modeling is its data-driven aspect. For instance, the parameters of the Gaussians (centre and variance) can be directly learnt from eye data via the expectation-maximization algorithm (Dempster et al. 1977), and the optimal number of Gaussian can be determined via a criterion such as the Bayesian Information Criterion, which penalizes the likelihood of models with too many parameters. To introduce the temporal component of gaze behavior in the approach, a few authors used hidden Markov models (HMMs), which capture the percentage of transitions from one region of interest (*state* of the model) to another (Chuk et al., 2017, 2014; Haji-Abolhassani & Clark, 2014; Coutrot et al., 2016). HMM parameters can be directly learnt from eye data via maximum likelihood estimation. For more details on HMM computation, cf. *Hidden Markov models* section. HMMs are data-driven, contain temporal information, and do not require high-quality eye data. Nevertheless, they are easily interpretable only with stimuli featuring clear regions of interest (cf. *Inferring observer characteristics from eye data* section). This gaze representation can be made even more compact with Fisher vectors, which are a concatenation of normalized GMM or HMM parameters into a single vector (Kanan et al. 2015). Although rich in information, these vectors are not intuitively interpretable. For a review of eye movement modeling with Markov processes, we refer the reader to Boccignone’s thorough introduction (Boccignone 2015). Some studies in the field of Biometry and gaze-based human identification propose a graph representation (Rigas et al. 2012; Cantoni et al. 2015; Galdi et al. 2016). For instance, in (Cantoni et al. 2015), the authors subdivided the clouds of fixation points with a grid to build a graph representing the gaze density, and fixation durations within each cell, and the transition probabilities between cells. Finally, spatial point processes constitute a probabilistic way of modeling gaze spatial distribution. They allow to jointly model the influence of different spatial covariates such as viewing biases or bottom-up saliency on gaze spatial patterns (Barthelmé et al. 2013; Engbert et al. 2015; Ylitalo et al. 2016). Their main drawback is that the temporal dimension is not taken into account.

### Contributions

The aim of this paper is to provide a ready-made solution for gaze modeling and classification, as well as an associated Matlab toolbox: *SMAC with HMM* (**S**canpath **M**odeling **A**nd **C**lassification with **H**idden **M**arkov **M**odels). Our approach is based on hidden Markov models (HMMs). It integrates influences stemming from top-down mechanisms, bottom-up mechanisms, and viewing biases into a single model. It answers to three criteria. First, it encapsulates the dynamic dimension of gaze behavior. Visual exploration is inherently dynamic: we do not average eye movements over time. Second, it encapsulates the individualistic dimension of gaze behavior. As mentioned in the introduction, visual exploration is a highly idiosyncratic process. As such, we want to model gaze in a data-driven fashion, learning parameters directly from eye data. Third, our approach is visually meaningful and intuitive. The rise of low-cost eye-tracking will enable a growing number of researchers to record and include eye data in their studies (Krafka et al. 2016). We want our model to be usable by scientists from all backgrounds. Our method works with any eye-data sampling frequency and do not require other input than gaze coordinates.

This paper is structured as follows. First, we formally describe HMMs in the context of gaze behavior modeling, and present our open-source toolbox. Then, we illustrate the strength and versatility of this approach by using HMM parameters to infer observers-related and stimuli-related characteristics from two very different datasets. Finally, we discuss some limitations of our framework.

## Methods

### Hidden Markov models for eye movement modeling

#### Definitions

HMMs model data varying over time, and can be seen as generated by a process switching between different phases or states at different time points. They are widely used to model Markov processes in fields as varied as speech recognition, genetics, or thermodynamics. Markov processes are memory-less stochastic processes: the probability distribution of the next state only depends on the current state and not on the sequence of events that preceded it. The adjective *hidden* means that a state is not directly observable. In the context of eye movement modeling, it can be inferred from the association between the assumed hidden state (region of interest - or ROI - of the image) and the observed data (eye positions). Here we follow the approach used in Chuk et al. (2014). More specifically, the emission densities, i.e., the distribution of fixations in each ROI, are modeled as two-dimensional Gaussian distributions. The transition from the current hidden state to the next one represents a saccade, whose probability is modeled by the transition matrix of the HMM. The initial state of the model, i.e., the probability distribution of the first fixation, is modeled by the prior values. To summarize, an HMM with K hidden states is defined by

1. $\mathcal {N}_{i}(m_{i},{\Sigma }_{i})_{i \in [1..K]}$, the Gaussian emission densities, with *m*_*i*_ the center and Σ_*i*_ the covariance of the *i* ^*th*^ state emission.
2. $A=(a_{ij})_{(i,j)\in [1..K]^{2}}$ the transition matrix, with *a*_*ij*_ the probability of transitioning from state *i* to state *j*.
3. $({p^{0}_{i}})_{i\in [1..K]}$ the priors of the model.

**Fig. 2:**
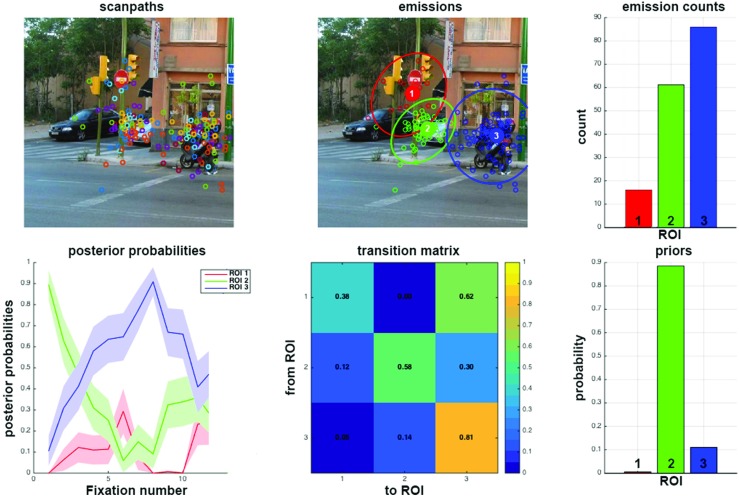
SMAC with HMM toolbox plot Three-state HMM modeling 19 scanpaths on an image from Koehler’s dataset. Scanpaths: fixation points of the same color belong to the same observer. Emissions: three states have been identified. Emission counts: number of fixations associated with each state. Posterior probabilities: temporal evolution of the probability of being in each state. *Shaded error bars* represent standard error from the mean. Transition matrix: probability of going from state (or region of interest) i to j, with (*i*,*j*) ∈ [1..3]^2^. Priors: initial state of the model

**Fig. 3:**
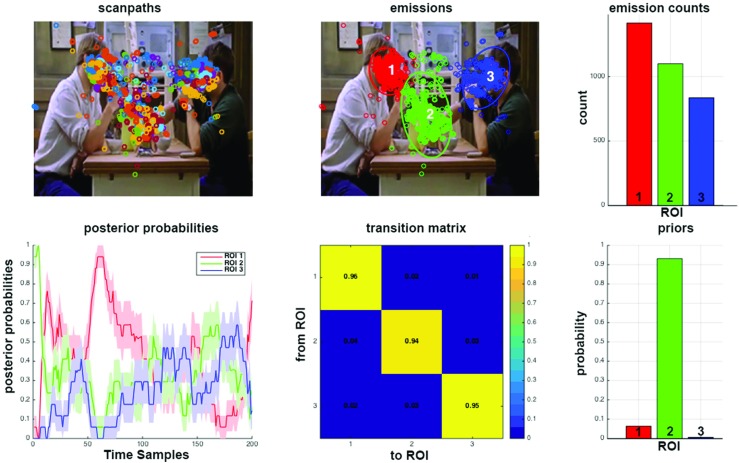
SMAC with HMM toolbox plot Three-state HMM modeling 19 scanpaths recorded on a video from Coutrot’s dataset. Scanpaths: eye positions of the same color belong to the same observer. Emissions: Three states have been identified. Emission counts: number of eye positions associated with each state. Posterior probabilities: temporal evolution of the probability of being in each state. *Shaded error bars* represent standard error from the mean. Transition matrix: probability of going from state (or region of interest) i to j, with (*i*,*j*) ∈ [1..3]^2^. Priors: initial state of the model

Figure 2 represents 19 scanpaths modeled by a single HMM. Scanpaths consisted of sequences of fixation points (on average, three fixations per second). Figure 3 is similar, but scanpaths consisted of eye positions time-sampled at 25 Hz. The sampling frequency impacts on the transition matrix coefficients: the higher the frequency, the closer to one the diagonal coefficients. Note that using time-sampled eye positions allows taking into account fixation durations.

#### Variational approach

A critical parameter is K, the number of state. For the approach to be as data-driven as possible, this value must not be determined a priori but optimized according to the recorded eye data. This is a problem since traditional maximum likelihood methods tend to give a greater probability for more complex model structures, leading to overfitting. In our case, a HMM with a great number of states might have a high likelihood but will be hard to interpret in term of ROI, and hard to compare to other HMMs trained with other sets of eye positions. The variational approach to Bayesian inference enables simultaneous estimation of model parameters and model complexity (McGrory and Titterington 2009). It leads to an automatic choice of model complexity, including the number of state K (see also Chuk et al., 2014, 2017).

#### Learning HMM from one or several observers

**Fig. 4:**
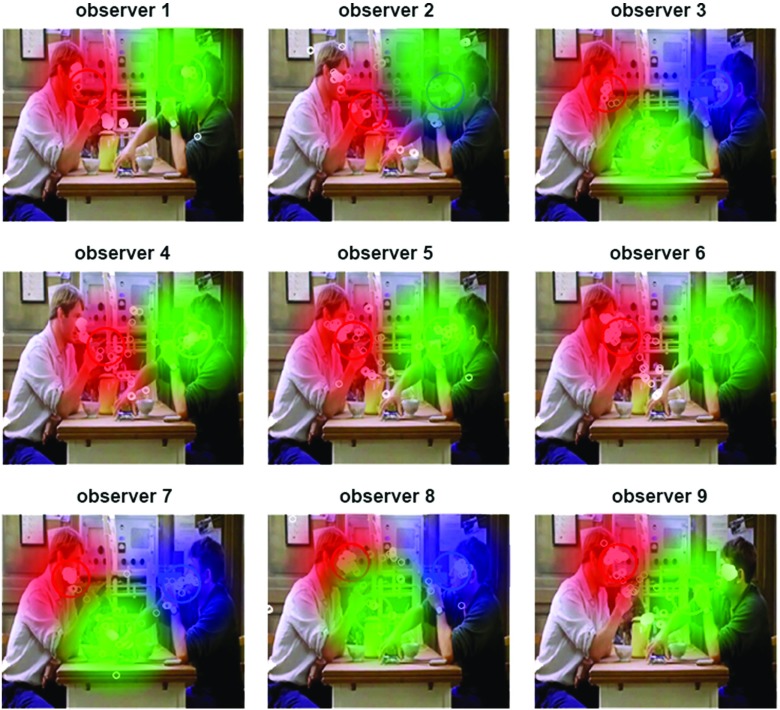
SMAC with HMM toolbox plot One HMM for each of nine scanpaths recorded on a video from Coutrot’s dataset. Maximum state number $K^{\max }=3$. *Small white circles* represent observer’s eye positions, *red*, *green*, and *blue* distributions represent HMM states. Covariance matrices have been tied to produce similar circular distributions

Two different approaches can be followed. An HMM can be learned from a group of scanpaths, as depicted in Figs. 2 and 3. This is useful to visualize and compare the gaze behavior of two different groups of observers, in two different experimental conditions for instance. It is also possible to learn one HMM per scanpath to investigate individual differences or train a gaze-based classifier, as depicted Fig. 4. In the following, we focus on the last approach. To link the HMM states learned from eye data to the actual ROI of the stimuli, we sort them according to their emissions’ center, from left to right. This allows comparing HMM learned from different scanpaths.

#### Toolbox

For more information, please refer to the *SMAC with HMM* toolbox manual in Supporting Information. The toolbox is available online at http://antoinecoutrot.magix.net/public/index.html.

### Classification from HMM parameters

A variety of classification methods have been used in the gaze-based inference literature, including discriminant analysis (linear or quadratic) (Greene et al. 2012; Tseng et al. 2013; Kardan et al. 2015; French et al. 2016; Coutrot et al. 2016), support vector machine (Lagun et al. 2011; Greene et al. 2012; Zelinsky et al. 2013; Tseng et al. 2013; Kanan et al. 2014; Lemonnier et al. 2014; Tavakoli et al. 2015; Borji et al. 2015; Wang et al. 2015; Kanan et al. 2015; Mills et al. 2015; French et al. 2016), naïve Bayes (Mercer Moss et al., 2012; Borji et al., 2015, Kardan et al., 2015, Mills et al., 2015), boosting classifiers (ADABoost, RUSBoost) (Borji and Itti 2014; Boisvert and Bruce 2016), Clustering (mean-shift, k-means, DBSCAN) (Rajashekar et al. 2004; Kang and Landry 2015; Engbert et al. 2015; Haass et al. 2016), random forests (Mills et al. 2015; Boisvert and Bruce 2016), and maximum likelihood estimation (Kanan et al. 2015; Coutrot et al. 2016). See (Boisvert and Bruce 2016) for a review. As stated in the Contributions section, this paper aims to provide an intuitive and visually meaningful method for gaze-based classification. We will focus on discriminant analysis as it includes both a predictive and a descriptive component: it is an efficient classification method, and it provides information on the relative importance of the variables (here, gaze features) used in the analysis. Let $g \in \mathbb {R}^{k}$ be a k-dimensional gaze feature vector and *G* *C* = {*g*_*i*_,*c*_*j*_}_*i*∈[1..*N*];*j*∈[1..*M*]_ be a set of *N* observations labeled by *M* classes. *n*_*j*_ is the number of observations in class *j*. Observations are the gaze features used to describe recorded eye data (here, HMM parameters). Classes can represent any information about the stimuli or the observers (e.g., task at hand, experimental condition, etc.).

#### Discriminant analysis

Discriminant analysis combines the *k* gaze features to create a new feature-space optimizing the separation between the *M* classes. Let *μ*_*j*_ be the mean of class *j* and *W*_*j*_ its variance-covariance matrix. The goal is to find a space where the observations belonging to the same class are as close as possible to each other, and as far away as possible from observations belonging to other classes. First, *g* is normalized to unit standard deviation and zero mean. The intra-group dispersion matrix *W* and the inter-group variance-covariance matrix *B* are defined by

1 $$ W=\frac{1}{N}\sum\limits_{j=1}^{M} n_{j} \times W_{j} \hspace{0.2cm}\text{and} \hspace{0.2cm} B = \frac{1}{N}\sum\limits_{j=1}^{M} n_{j}(\mu_{j}-\mu)'(\mu_{j}-\mu) $$

with *μ* the global mean. The symbol ^′^ represents the transposition. The Eigen vectors **u** of the new space maximize the expression

2 $$ {arg\,max}_{u} (\frac{u^{\prime}Bu}{u^{\prime}(W+B)u} ) $$

The absolute values of the coefficients of **u** provide information on the relative importance of the different gaze features to separate the classes: the higher the value, the more important the corresponding feature.

#### Classification

The method is general, but for the sake of clarity, let’s focus on a LDA-based two-classes classification only. Let *y*_1_ and *y*_2_ be the respective projections of class 1 and class 2 average on **u**.

3 $$ y_{1} = u^{\prime}\mu_{1} \hspace{0.2cm}\text{and} \hspace{0.2cm} y_{2} = u^{\prime}\mu_{2} $$

Let *g*_0_ be the new observation we want to classify and *y*_0_ = *u* ^′^ *μ*_0_ the projection of its mean on **u**. The classification consists in assigning *g*_0_ to the class whose average it is closest to along **u**, i.e.,

4 $$ \text{ L{\kern-.23pt}e{\kern-.23pt}t{\kern-.23pt}'{\kern-.23pt}s{\kern-.23pt} a{\kern-.23pt}s{\kern-.23pt}s{\kern-.23pt}u{\kern-.23pt}m{\kern-.23pt}e{\kern-.23pt} } y_{1}\!>\!y_{2}. g_{0} \text{ i{\kern-.23pt}s{\kern-.23pt} a{\kern-.23pt}s{\kern-.23pt}s{\kern-.23pt}i{\kern-.23pt}g{\kern-.23pt}n{\kern-.23pt}e{\kern-.23pt}d{\kern-.23pt} t{\kern-.23pt}o{\kern-.23pt} c{\kern-.23pt}l{\kern-.23pt}a{\kern-.23pt}s{\kern-.23pt}s{\kern-.23pt} 1{\kern-.23pt} i{\kern-.23pt}f{\kern-.23pt}} \hspace{0.1cm} \!y_{0\!}>\!\frac{y_{1}\,+\, y_{2}}{2} $$

We follow a leave-one-out approach: at each iteration, one observation is taken to test, and the classifier trained with all the others. The correct classification rate is then the number of iteration where the class is correctly guessed divided by *N*, the total number of iteration.

#### Application to gaze-based inference

**Fig. 5:**
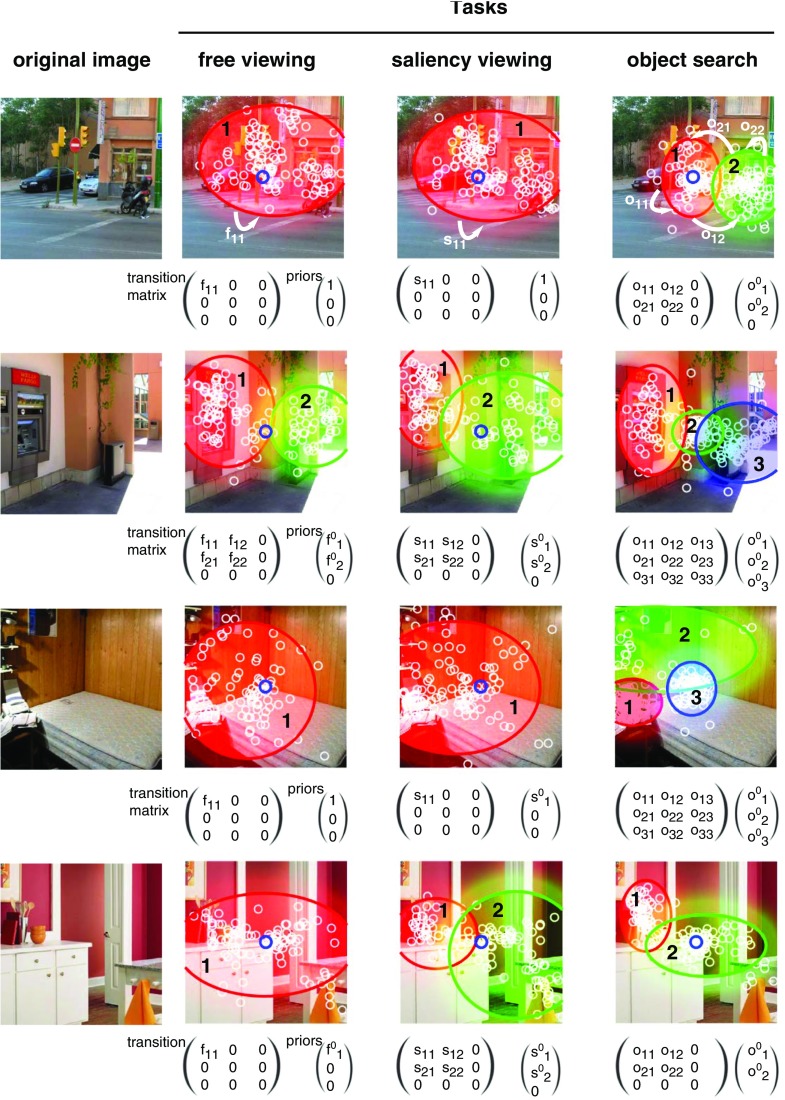
Hidden Markov models for four images and three tasks. For each image and each task, we train one HMM with the eye data of one observer. *Small white circles* represent the fixations of all observers following the same task. HMMs are made of states represented by Gaussian pdf (*red*, *green*, and *blue*), a transition matrix and priors. The optimal number of state has been determined by Bayesian variational approach

Here, the gaze feature vector *g* is made of HMM parameters.

5 $$ \textbf{g} \,=\, [({p_{i}^{0}})_{i \in [1..K]}, (a_{ij})_{(i,j)\in [1..K]^{2}}, (m_{i})_{i\in [1..K]},({\Sigma}_{i})_{i \in [1..K]}] $$

with (*p*_*i*_) the priors, (*a*_*ij*_) the transition matrix coefficients, (*m*_*i*_) and (Σ_*i*_) the center and covariance matrix coefficients of the Gaussian emissions. *K* represents the number of state used in the HMM. As presented in the previous section, this number is determined by a variational approach and can change from one observation to the other. In order for *g* to have the same dimensionality for all observations, we define *K* ^max^ as the highest number of states across all observations. For observations where *K* < *K* ^max^, we pad their gaze feature vector with zeros, introducing ”ghost states”. See for instance first row of Fig. 5, where *K* ^max^ = 3. In the free viewing and saliency viewing tasks, only one state is used, the coefficients corresponding to the other ones are set to zero. For the object search task, two states are used, the coefficients of the last one are set to zero.

#### Regularization

A problem can appear if gaze feature vectors are padded with too many zeros, or if the dimension of **g** exceeds the number of observations *N*. In that case, the intra-group dispersion matrix *W* is singular and therefore cannot be inverted: Eigen vectors **u** cannot be computed. To solve the problem, two solutions can be adopted. The first one is to simply reduce the dimensionality of **g** with a principal component analysis, keeping only the *P* < *N* first principal components. The second one is to use a regularized discriminant analysis approach (rDA) which uses (1 − *λ*)*W* + *λ* *I* instead of *W*, with small *λ* called the shrinkage estimator.

## Results

We illustrate the versatility of our approach with two very different public datasets. In the first one, we model gaze behavior on 800 still natural scene images, and infer an observer-related characteristic: the task at hand. In the second one, we model gaze behavior on 15 conversational videos, and infer a stimuli-related characteristic: the presence or absence of original soundtrack.

### Inferring observer characteristics from eye data

#### Koehler’s dataset

This dataset was originally presented in (Koehler et al. 2014) and is freely available online^1^. It consists of 158 participants split into three tasks: free viewing, saliency search task, and cued object search task. Participants in the saliency search condition were instructed to determine whether the most salient object or location in an image was on the left or right half of the image. Participants in the cued object search task were instructed to determine whether a target object was present in a displayed image. Stimuli consisted of 800 natural scenes pictures, comprising both indoor and outdoor locations with a variety of sceneries and objects. Images were centrally displayed on a gray background for 2000 ms, and had a resolution of 15 × 15 degrees of visual angle. Every trial began with an initial fixation cross randomly placed either centered, 13 degrees left of center or 13 degrees right of center. Eye data was recorded with an Eyelink 1000 monitoring gaze position at 250 Hz.

#### HMM computation

We trained one HMM per scanpath, i.e., one HMM per participant and per image. We set *K* ^max^ = 3. Higher values of *K* ^max^ have been tried, but in most instances the variational approach selected models with *K* ≤ 3. As a minimum of four points are needed to compute three-state HMM, scanpath with fewer than four fixations have been discarded from the analysis. We did not set a maximum number of fixations. Four representative examples are given in Fig. 5.

#### Task classification

**Fig. 6:**
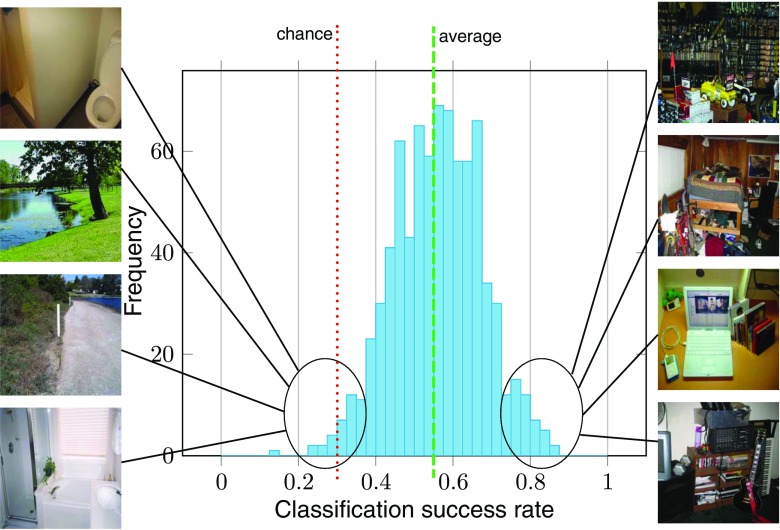
Task classification success rate histogram. The average success rate is .559, significantly above chance (.33, permutation test, *p* < 0.001). Each sample of this distribution corresponds to the mean classification rate for a given image. We show eight images drawn from the left and right tail of the distribution. Images with good task classification rate contain more salient objects. On the contrary, tasks while viewing images without particularly salient objects are harder to classify

**Fig. 7:**
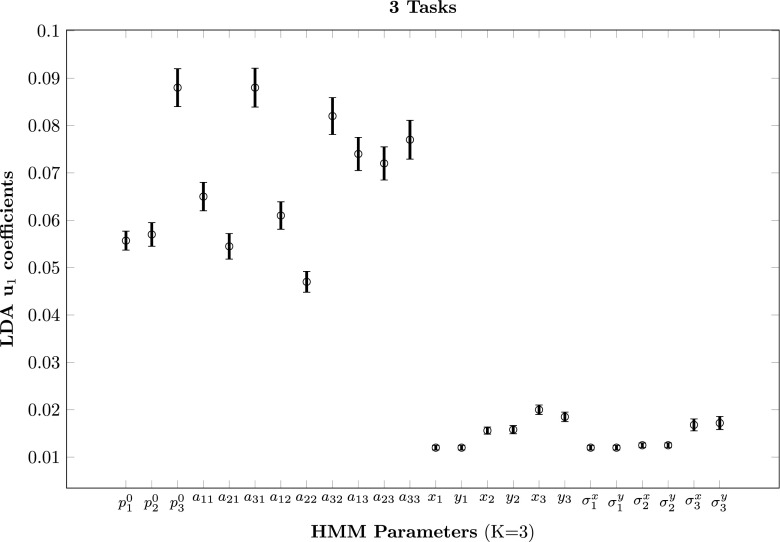
LDA first eigenvector coefficients (absolute values, unity-sum constraint). $({p_{i}^{0}})_{i \in [1..K]}$ represent the priors, $\protect (a_{ij})_{i,j \in [1..K]^{2}}$ represent the transition matrix coefficients, (*x*_*i*_,*y*_*i*_)_*i*∈[1..*K*]_ represent the center of the Gaussian states and $\protect ({\sigma _{i}^{x}},{\sigma _{i}^{y}})_{i \in [1..K]}$ represent their variance along the *x* and *y* axis. The higher the coefficient, the more important the corresponding parameter to separate the classes. These coefficients optimize the separation between the three tasks in Koehler’s data. The maximum number of state is K = 3

Each scanpath is described by a 24-dimensional vectors *g*: since *K* ^max^ = 3, there are three priors, 3 × 3 transition matrix coefficients, 3 × 2 Gaussian center coordinates and 3 × 2 Gaussian variance coefficients along the *x* and *y* axis. These parameters have different magnitudes, so *g* is normalized to unit standard deviation and zero mean. Regularized linear discriminant analysis is then used for classification. Since ’ghost’ states might be involved (for models where *K* < *K* ^max^), we had to regularize the training matrix. We took (1 − *λ*)*W* + *λ* *I* instead of *W*, with *λ* = 1*e* − 5. We followed a leave-one-out approach: at each iteration, we trained the classifier with all but one scanpath recorded on a given image, and tested with the removed scanpath. This led to an average correct classification rate of 55.9% (min = 12.9%, max = 87.2%, 95% confidence interval (CI) = [55.1% 56.7%]). See Fig. 6 for the distribution of classification rates across stimuli. This classifier performs significantly above chance (which is 33%). To test the significance of this performance, we ran a permutation test. We randomly shuffled the label of the class (the task) for each observation and computed a ’random classification rate’. We repeated this procedure 1e5 times. A *p* value represents the fraction of trials where the classifier did as well as or better than the original data. In our case, we found *p* < 0.001. In Fig. 7, we show the absolute average values of the coefficients of the first LDA eigenvector, with unity-sum constraint. The higher the coefficients, the more important they are to separate the three classes. First, we notice that the priors and the transition matrix coefficients play a bigger role than Gaussian parameters. Then, we see that all the parameters linked to the third state are higher than the other ones. We computed the average number of ’real’ states for all scanpaths in the three tasks. We found that during the search task, scanpaths have significantly more ’real’ states (*M* = 2.26, 95% CI = [2.25 2.27]) than during free viewing or saliency viewing (both *M* = 2.12, 95% CI = [2.11 2.13]).

#### Is the method equally efficient with all visual content?

Correct classification rates have a Gaussian-shaped distribution across stimuli, ranging from 10 to 80%, see Fig. 6. Why is task classification more efficient with some images than with others? We hypothesize that in order to have a high correct classification rates, images must contain various regions of interest. If there is no region of interest (e.g., a picture of a uniform sky), observers’ exploration strategies might be too random for the classifier to capture systematic patterns diagnostic of a given class. If the image only contains one salient object (e.g., a red ball on a beach), observers’ exploration strategies might be too similar: everyone would stay focused on the only region of interest, and the classifier would fail for the same reason. To test this hypothesis, we looked at the correlation between the number of regions of interest and the image correct classification score. To compute the number of regions of interest, for each image, we computed its bottom-up saliency map with the attention based on information maximization (AIM) and the adaptive whitening saliency (AWS) models (Bruce and Tsotsos 2006; Garcia-Diaz et al. 2012). We chose AIM and AWS because they provide good saliency estimation and the least spatially biased results, rendering them suitable for tasks in which there is no information about the underlying spatial bias of the stimuli (Wloka and Tsotsos 2016). Each saliency map is thresholded to a binary image. The number of regions of interest (or ’salient blobs’) in the binary map is the number of connected components (*bwlabel* Matlab function). We found a positive significant Pearson’s correlation between the number of salient objects and the classification score both for AIM (*r* = 0.14,*p* < 0.001) and AWS (*r* = 0.1,*p* = 0.01). This means that images with higher correct classification rates contain more salient objects. On the other hand, images without particularly salient objects are harder to classify.

### Inferring stimulus characteristics from eye data

#### Coutrot’s dataset

This dataset was originally presented in (Coutrot and Guyader 2014) and is freely available online^2^. It consists of 15 conversational videos split into auditory conditions: with or without original soundtrack. Videos featured conversation partners embedded in a natural environment, lasted from 12 to 30 s and had a resolution of 28 × 22.5 degrees of visual angle. Original soundtracks were made of conversation partners’ voice and environmental noises, non-original soundtracks were made of natural meaningless slowly varying sounds such as wind or rain sounds. Each video has been seen in each auditory condition by 18 different participants. Every trial began with an initial centered fixation cross. Eye data were recorded with an Eyelink 1000 monitoring gaze position at 1000 Hz.

#### HMM computation

**Fig. 8:**
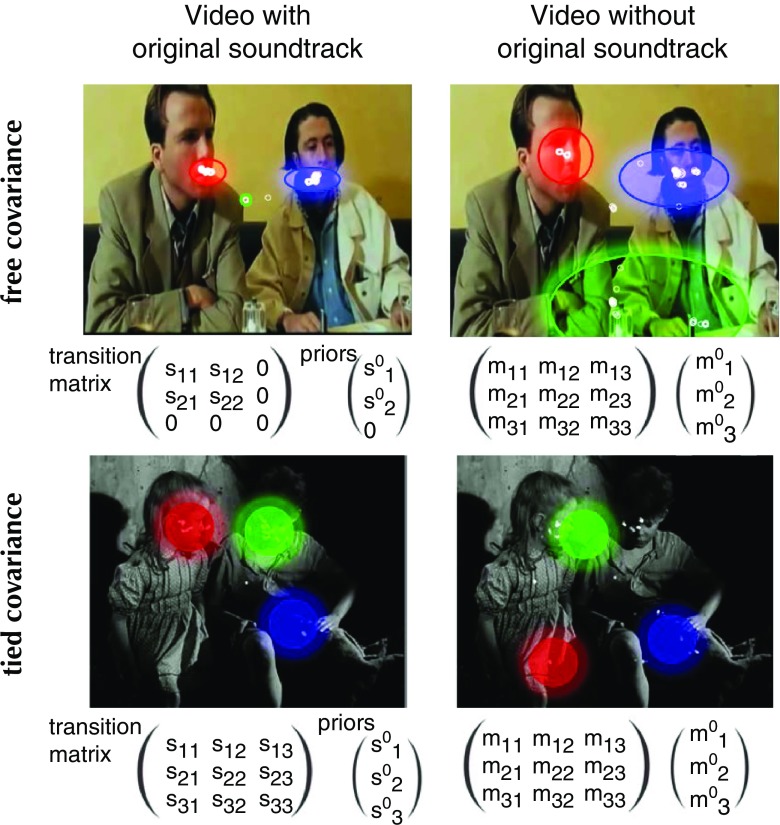
Hidden Markov models for two videos and two auditory conditions. For each video, we train one HMM with the eye data of one observer (*small white circles*) in each auditory condition (with or without the original soundtrack). HMMs are made of states represented by Gaussian pdf (*red*, *green*, and *blue*), a transition matrix and priors. The optimal number of states has been determined by Bayesian variational approach. The covariance of the HMM states on the first row is data-driven, while the one of the second rows has been tied to a circular distribution

We trained one HMM per scanpath, i.e., one HMM per participant and per video. HMM were trained with the average gaze positions of the 200 first frames of the video (8 s), i.e., with 200 gaze points. We set *K* ^max^ = 3. As with Koehler’s dataset, higher values of *K* ^max^ have been tried, but the variational approach selected models with *K* ≤ 3. On the first row of Fig. 8, we give an example where ROIs’ covariances are determined by the data. ROI’s covariance seems larger without than with the original soundtrack. To test this, we computed the average real state covariance for each HMM: $\bar {\sigma }=\sqrt {{\sigma ^{2}_{x}}+{\sigma ^{2}_{y}}}$. We indeed found a greater average covariance without (*M*=5568 pixels, 95% CI = [5049 6087]) than with (*M*=4400 pixels, 95% CI = [3859 4940]) the original soundtrack (two-sample *t* test: *p* = 0.002). On the second row of Fig. 8, we used a method called *parameter tying* to force a unique covariance matrix across all states (Rabiner 1989). A parameter is said to be tied in the HMMs of two scanpaths if it is identical for both of them. Tying covariances makes all emissions cover the same area. This can be useful when the size of the ROIs is similar and consistent across stimuli, which is the case in this dataset where faces are always the most salient objects. We chose $\sum = \left (\begin {array}{cc} 500&0\\ 0&500 \end {array} \right )$so state distributions are circles of the same size as conversation partners’ faces.

#### Stimuli classification

We followed the same approach as described for Koehler’s dataset, except that we have here two classes of auditory conditions. Using parameter tying and *K* ^max^ = 3, we achieve an average correct classification rate over all stimuli of 81.2% (min = 54.3%, max = 91.9%, 95% CI = [76.1% 86.3%]). This classifier performs significantly above chance (50%, permutation tests: *p* < 0.001).

### Comparison with other gaze features and classifiers

In this section, we compare the performance of our HMM-based gaze features with other gaze features used in the literature. As described in the introduction, gaze has been modeled in two ways: static (averaging eye movement parameters over time) and dynamic (representing gaze as a time series). We chose to compare our method with two widely popular representatives of each approach. *Static*: we use average fixation duration, standard deviation of the fixation duration distribution, saccade amplitude, standard deviation of the saccade amplitude distribution, eye position dispersion (within-subject variance), and the first five eye position coordinates, as in (Greene et al. 2012; Borji and Itti 2014; Kardan et al. 2015; Mills et al. 2015; Tavakoli et al. 2015). We can apply to these features the same classifiers as to our HMM-based features. We used linear discriminant analysis (LDA), support vector machine with linear kernel (SVM), relevance vector machine (RVM) and AdaBoost. RVM is similar to SVM but uses Bayesian inference to obtain parsimonious solutions for probabilistic classification (Tipping 2001). AdaBoost investigates non-linear relationships between features by combining a number of weak classifiers (here set at 100) to learn a strong classifier. It had been previously successfully used in visual attention modeling (Zhao and Koch 2012; Borji 2012; Boisvert and Bruce 2016). *Dynamic*: we use ScanMatch, designed to compare pairs of scanpath (Cristino et al. 2010). This method is based on the Needleman–Wunsch algorithm used in bioinformatics to compare DNA sequences. It compares scanpaths of each class with each other, within and between classes. Within-class comparisons should have higher similarity scores than between class comparisons. A k-mean clustering algorithm is used to classify each comparison to either the within or between-class group. We used Coutrot’s dataset as fixation durations and saccade amplitudes are not available in Koehler’s data. Moreover, it is a two-class classification problem (with or without original soundtrack), directly compatible with ScanMatch. We compared the performance of classifiers trained with static and dynamic features previously used in the literature, HMM spatial features (ROI center coordinates and covariance), HMM temporal features (priors and transition matrix coefficients), and HMM spatio-temporal features (both). Table 1 shows that the best results are achieved with LDA trained with HMM spatio-temporal features.

**Table 1:** Correct classification scores on Coutrot’s dataset, with different gaze features and classifiers

**Gaze features**	**LDA**	**SVM**	**RVM**	**AdaBoost**	**k-means**
**Static** (saccades & fixations parameters averaged over time)	52.4%	**56.7**%	**63.8**%	**57.6**%	n/a
**Dynamic** (ScanMatch scores)	n/a	n/a	n/a	n/a	**59.5**%
**HMM spatial** features (ROI mean + covariance)	**59.0**%	**57.4**%	**62.5**%	55.2%	n/a
**HMM temporal** features (priors + transition matrix)	50.3%	54.8%	**61.4**%	54.6%	n/a
**HMM spatio-temporal** features (priors + transition matrix + mean + covariance)	**81.2**%	**58.0**%	**58.7**%	54.8%	n/a

Scores significantly above chance are in bold (binomial test, *p* < 0.05 between 56% and 59%, *p* < 0.001 above 59%). Chance level is 50%

## Discussion

### Integrating bottom-up, top-down, and oculomotor influences on gaze behavior

Visual attention, and hence gaze behavior, is thought to be driven by the interplay between three different mechanisms: bottom-up (stimuli-related), top-down (observer-related), and spatial viewing biases (Kollmorgen et al. 2010). In this paper, we describe a classification algorithm relying on discriminant analysis (DA) fed with hidden Markov models (HMMs) parameters directly learnt from eye data. By applying it on very different datasets, we showed that this approach is able to capture gaze patterns linked to each mechanism.

### Bottom-up influences

We modeled scanpaths recorded while viewing conversational videos from Coutrot’s dataset. Videos were seen in two auditory conditions: with and without their original soundtracks. Our method is able to infer under which auditory condition a video was seen with an 81.2% correct classification rate (chance = 50%). HMMs trained with eye data recorded without the original soundtrack had ROIs with a greater average covariance than with the original soundtrack. This is coherent with previous studies showing that the presence of sound reduces the variability in observers’ eye movements (Coutrot et al. 2012), especially while viewing conversational videos (Foulsham and Sanderson 2013; Coutrot and Guyader 2014). This shows that HMMs are able to capture a bottom-up influence: the presence or absence of original soundtrack.

### Top-down influences

We also modeled scanpath recorded while viewing static natural scenes from Koehler’s dataset. Observers were asked to look at pictures under three different tasks: free viewing, saliency viewing, and object search. Our method is able to infer under which task an image was seen with a 55.9% correct classification rate (chance = 33.3%). HMMs are hence able to capture a top-down influence: the task at hand. This complements two previous studies that also successfully used HMMs to infer observer-related properties: observer’s gender during face exploration (Coutrot et al. 2016), and observer’s processing state during reading (Simola et al. 2008).

### Viewing biases

Looking at Fig. 5, we notice that in the search task there is often a greater number of fixations at the center of the stimuli than in the other tasks. This cluster of central fixations is clearly modeled by a third HMM state in the second and third images. On average across all stimuli, we found a higher number of “real” HMM states in the search task than in the free viewing or saliency viewing task (2.26 versus 2.12). Figure 7 indicates that LDA first eigenvector coefficients related to the third state are higher than the other ones. Having a “real” third component is hence one of the criterion used by the classifier as a good marker of the search task. Moreover, the posterior probabilities of the states displayed Fig. 2 indicate that this center bias is stronger at the beginning of the exploration. This corroborates the idea that the center of the image is an optimal location for early and efficient information processing, often reported in the literature as the *center bias* (Tatler 2007). Hence, HMMs are able to integrate influences stemming from top-down mechanisms (task at hand), bottom-up mechanisms (presence of original soundtrack), and viewing biases (center bias) in a single model of gaze behavior.

### Interpretability

The choice of both gaze features and classification algorithm is fundamental for efficient classification. A good illustration of this is Greene et al.’s reported failure to computationally replicate Yarbus’ seminal claim that the observers’ task can be predicted from their eye movement patterns (Greene et al. 2012). Using linear discriminant analysis and simple eye movement parameters (fixation durations, saccade amplitudes, etc.), they did not obtain correct classification rates higher than chance. In 2014, Borji et al. and Kanan et al. obtained positive results with the same dataset by respectively adding spatial and temporal information (Borji and Itti 2014; Kanan et al. 2014). They used non-linear classification methods such as k-nearest-neighbors (kNN), random undersampling boosting (RUSBoost) and Fisher kernel learning, and obtained correct classification rates significantly above chance. Going further, one can hypothesize that even higher correct classification rates could be reached using deep learning networks (DLN), which have proven unbeatable for visual saliency prediction (Bylinskii et al. 2015). However, boosting algorithms and DLN suffer from an important drawback: both rely on thousands of parameters, whose roles and weights are hard to interpret (although see (Lipton 2016)). Conversely, in addition to providing good correct classification rates, our approach is easy for users to understand and interpret. Our classification approach takes as input a limited number of identified and meaningful HMM parameters (priors, transition probability between learnt regions of interest, Gaussians center and covariance), and outputs weights, indicating the importance of the corresponding parameters in the classification process.

### Simplicity

In order to make gaze-based classification easily usable in as many contexts as possible, relying on simple features is essential. In a recent study, Boisvert et al. used Koehler’s dataset to classify observers’ task from eye data (Boisvert and Bruce 2016). They achieved a correct classification score of 56.37%, similar to ours (55.9%). They trained a random forest classifier with a combination of gaze-based (fixation density maps) and image-based features. They convolved each image with 48 filters from the Leung-Malik filter bank corresponding to different spatial scales and orientations (Leung and Malik 2001), and extracted the response of each filter at each eye position. They also computed histogram of oriented gradients from every fixated location, as well as a holistic representation of the scene based on the Gist descriptor (Oliva and Torralba 2006). This approach is very interesting, as it allows assessing the role of specific features or image structure at fixated locations. However, computing such features can be computationally costly, and even impossible if the visual stimuli are not available. On the other hand, our approach only relies on gaze coordinates, either fixations or eye positions sampled at a given frequency.

### Limitations

Our approach suffers from a number of limitations. First, HMMs are dependent on the structure of the visual stimuli. In order to have a meaningful and stable model, stimuli must contain regions of interest (ROIs). For instance, modeling the visual exploration of a uniform landscape is difficult as nothing drives observers’ exploration: the corresponding HMM would most likely have a single uninformative central state. This is illustrated by the distribution of correct classification rates across stimuli, in Fig. 6. We showed a positive correlation between the number of ROIs and the image correct classification rates. This means that in order for different gaze patterns to develop—and to get captured by the model—visual stimuli must feature a few salient regions. Another consequence of the dependence on visual content is the difficulty-to-aggregate eye data recorded while viewing different stimuli. It is possible when the stimuli share the same layout, or have similar ROIs. For instance, a recent study used eye data of observers looking at different faces to train a single HMM (Coutrot et al. 2016). This was possible since faces share the same features and can be ’aligned’ to each other; but this would not be possible with Koehler’s dataset, as it is made of diverse natural scenes featuring ROIs from various sizes at various locations. The same difficulties arise when considering dynamic stimuli. Here we were able to use Coutrot’s conversational videos, as conversation partners remain at the same position through time. However, it would be more complicated with videos where ROIs move across time. A solution would be to train models on time windows small enough for the ROIs not to move too much. A serious drawback would be the reduced number of eye data available within each time window. Another possibility would be to use computer vision tools to detect and track ROIs as they move. For instance, it is possible to parse a video into supervoxels representing homogeneous regions through time, and use them as HMM nodes (Rai et al. 2016). This approach can also be used to improve the comparison of different HMMs with many states. Indeed, the greater the number of states, the harder it is to compare two HMMs trained from different observers. For instance, in a scene with two conversation partners, a HMM with three states is likely to capture the head of the two speakers, and the background (as in Fig. 8). When increasing the number of states, HMMs will capture less significant regions that are likely to vary a lot from one observer to the other, making the comparison between HMMs challenging. Detecting and tracking ROIs would allow a direct comparison of the states, for instance based on their semantics. But by introducing stimuli information, this increases the complexity of the model (see previous paragraph). Finally, even though our model takes into account top-down, bottom-up, and oculomotor influences, in some cases it might not be enough. There are a number of contexts where visual attention is strongly biased by what happened in the past (e.g., reward and selection history), which is not explicitly taken into account by the three aforementioned mechanisms (Awh et al. 2012). However, HMM framework lends itself well to memory effect modeling. For instance, in (Hua et al. 2015), the authors proposed a memory-guided probabilistic visual attention model. They constructed a HMM-like conditional probabilistic chain to model the dynamic fixation patterns among neighboring frames. Integrating such a memory module into our model could improve its performance in a variety of situations, for instance when watching a full-length movie, where prior knowledge builds up with time.

## Conclusions

We have presented a scanpath model that captures the dynamic and individualistic components of gaze behavior in a data-driven fashion. Its parameters reveal visually meaningful differences between gaze patterns and integrate top-down, bottom-up, and oculomotor influences. We also provide *SMAC with HMM*, a turnkey Matlab toolbox requiring very simple inputs. This method can be used by a broad range of scientists to quantify gaze behavior. A very promising application would be to integrate our approach in visual attention saccadic models. Like saliency models, saccadic models aim to predict the salient areas of our visual environment. However, contrary to saliency models they also must output realistic visual scanpaths, i.e., displaying the same idiosyncrasies as human scanpaths (Le Meur and Liu 2015; Le Meur and Coutrot 2016). Training HMM with a specific population of observers (e.g., experts vs. novices) would allow tailoring HMM-based saccadic model for this population. In the same vein, it would also be possible to tailor saccadic models for a specific type of stimuli, or for observers having specific oculomotor biases (e.g., patients).

## Footnotes

1
https://labs.psych.ucsb.edu/eckstein/miguel/research_pages/saliencydata.html

2
http://antoinecoutrot.magix.net/public/databases.html
